# 3,3′-[1,4-Phenyl­enebis(methyl­ene)]­bis­(1-propyl­benzimidazolium) dichloride dihydrate

**DOI:** 10.1107/S1600536812007738

**Published:** 2012-02-24

**Authors:** Rosenani A. Haque, Muhammad Adnan Iqbal, Safaa A. Ahmad, Tze Shyang Chia, Hoong-Kun Fun

**Affiliations:** a School of Chemical Sciences, Universiti Sains Malaysia, 11800 USM, Penang, Malaysia; b Department of Chemistry, College of Education Samarra, University of Tikrit, Tikrit 43001, Iraq; c X-ray Crystallography Unit, School of Physics, Universiti Sains Malaysia, 11800 USM, Penang, Malaysia

## Abstract

The asymmetric unit of the title compound, C_28_H_32_N_4_^2+^·2Cl^−^·2H_2_O, contains half of a 3,3′-[1,4-phenyl­enebis(methyl­ene)]bis­(1-propyl­benzimidazolium) cation, one chloride anion and one water mol­ecule. The complete cation is generated by a crystallographic inversion center. The central benzene ring forms a dihedral angle of 66.06 (11)° with its adjacent benzimidazolium ring system. In the crystal, the cations, anions and water mol­ecules are linked by O—H⋯Cl, C—H⋯O and C—H⋯Cl hydrogen bonds into a three-dimensional network. The crystal packing is further stabilized by π–π inter­actions, with centroid–centroid distances of 3.5561 (15) and 3.6708 (15) Å.

## Related literature

For details and applications of benzimidazole derivatives, see: Narasimhan *et al.* (2012 ▶). For related structures, see: Haque *et al.* (2011 ▶, 2012 ▶); Iqbal *et al.* (2012 ▶). For reference bond lengths, see: Allen *et al.* (1987 ▶). For the stability of the temperature controller used for data collection, see: Cosier & Glazer (1986 ▶).
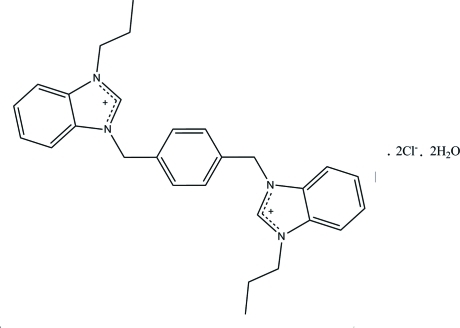


## Experimental

### 

#### Crystal data

C_28_H_32_N_4_^2+^·2Cl^−^·2H_2_O
*M* *_r_* = 531.51
Monoclinic, 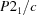

*a* = 8.1177 (5) Å
*b* = 9.1042 (5) Å
*c* = 18.3548 (11) Å
β = 94.323 (2)°
*V* = 1352.66 (14) Å^3^
*Z* = 2
Mo *K*α radiation
μ = 0.27 mm^−1^
*T* = 100 K
0.47 × 0.23 × 0.14 mm

#### Data collection

Bruker SMART APEXII CCD area-detector diffractometer
Absorption correction: multi-scan (*SADABS*; Bruker, 2009 ▶) *T*_min_ = 0.882, *T*_max_ = 0.962
11942 measured reflections
3085 independent reflections
2660 reflections with *I* > 2σ(*I*)
*R*_int_ = 0.053

#### Refinement

*R*[*F* ^2^ > 2σ(*F* ^2^)] = 0.061
*wR*(*F* ^2^) = 0.196
*S* = 1.08
3085 reflections
172 parameters
H atoms treated by a mixture of independent and constrained refinement
Δρ_max_ = 0.86 e Å^−3^
Δρ_min_ = −0.48 e Å^−3^

### 

Data collection: *APEX2* (Bruker, 2009 ▶); cell refinement: *SAINT* (Bruker, 2009 ▶); data reduction: *SAINT*; program(s) used to solve structure: *SHELXTL* (Sheldrick, 2008 ▶); program(s) used to refine structure: *SHELXTL*; molecular graphics: *SHELXTL*; software used to prepare material for publication: *SHELXTL* and *PLATON* (Spek, 2009 ▶).

## Supplementary Material

Crystal structure: contains datablock(s) global, I. DOI: 10.1107/S1600536812007738/is5076sup1.cif

Structure factors: contains datablock(s) I. DOI: 10.1107/S1600536812007738/is5076Isup2.hkl

Supplementary material file. DOI: 10.1107/S1600536812007738/is5076Isup3.cml

Additional supplementary materials:  crystallographic information; 3D view; checkCIF report

## Figures and Tables

**Table 1:** Hydrogen-bond geometry (Å, °)

*D*—H⋯*A*	*D*—H	H⋯*A*	*D*⋯*A*	*D*—H⋯*A*
O1*W*—H1*W*1⋯Cl1	0.93 (4)	2.29 (4)	3.200 (3)	166 (4)
O1*W*—H2*W*1⋯Cl1^i^	0.84 (5)	2.30 (4)	3.130 (3)	170 (4)
C1—H1*A*⋯Cl1^ii^	0.95	2.81	3.677 (3)	153
C4—H4*B*⋯Cl1^i^	0.99	2.77	3.741 (3)	167
C6—H6*A*⋯Cl1^ii^	0.95	2.76	3.691 (3)	165
C11—H11*A*⋯O1*W*	0.95	2.14	3.059 (4)	163
C12—H12*A*⋯Cl1	0.99	2.79	3.747 (3)	164
C12—H12*B*⋯Cl1^iii^	0.99	2.75	3.740 (3)	175

Symmetry codes: (i) 
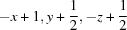
; (ii) 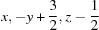; (iii) 
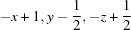
.

**Fig. 1.**
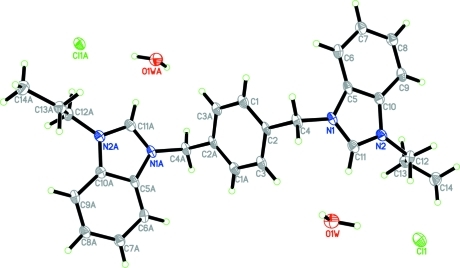
The molecular structure of the title compound with atom labels and 50% probability displacement ellipsoids. Atoms with suffix A were generated by symmetry code -x, -y + 2, -z.

**Fig. 2.**
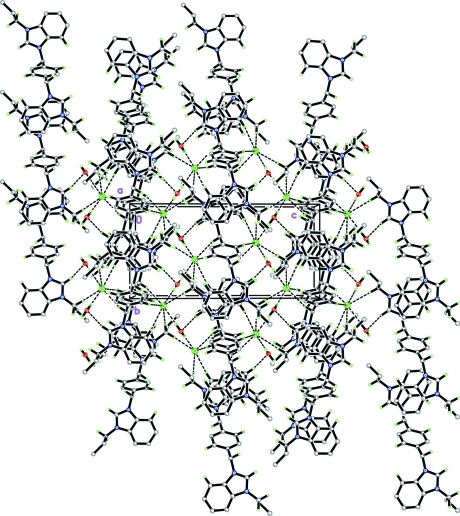
The crystal packing of the title compound. Intermolecular hydrogen bonds are shown as dashed lines. H atoms not involved in the hydrogen bonds have been omitted for clarity.

## supplementary crystallographic information

### Comment

Benzimidazole constituted compounds have diverse biological and clinical applications (Narasimhan *et al.*, 2012). Previously, we have reported crystal structures of *ortho*-xylyl linked *bis*-benzimidazolium salts with heptyl (Haque *et al.*, 2011), propyl (Iqbal *et al.*, 2012), and ethyl (Haque *et al.*, 2012) substitutions. In this report, we describe the crystal structure of a *para*-xylyl linked *bis*-benzimidazolium salt with propyl substitutions.

The molecular structure of the title compound is shown in Fig. 1. The asymmetric unit of the title compound, C_28_H_32_N_4_^2+^.2Cl^-^.2H_2_O, consists of one half-molecule of 3,3'-[1,4-phenylenebis(methylene)]bis(1-propyl-benzimidazolium) cation, one chlorine anion and one water molecule. The complete cation is generated by a crystallographic inversion center (-*x*, -*y* + 2, -*z*). The central benzene ring (C1–C3/C1A–C3A) forms a dihedral angle of 66.06 (11)° with its adjacent benzimidazolium ring (N1/N2/C5–C11) [maximum deviation = 0.031 (2) Å at atom C5]. Bond lengths (Allen *et al.*, 1987) and angles are within normal ranges and are comparable to related structures (Haque *et al.*, 2011,2012; Iqbal *et al.*, 2012).

In the crystal structure, (Fig. 2), the cations, anions and water molecules are linked by intermolecular O1W—H1W1···Cl1, O1W—H2W1···Cl1, C1—H1A···Cl1, C4—H4B···Cl1, C6—H6A···Cl1, C11—H11A···O1W, C12—H12A···Cl1, and C12—H12B···Cl1 hydrogen bonds (Table 1) into a three-dimensional network. The crystal packing is further stabilized by π–π interactions with *Cg*1···*Cg*3 (1-*x*, 1-*y*, -*z*) distance = 3.5561 (15) Å and *Cg*3···*Cg*3 (1-*x*, 1-*y*, -*z*) distance = 3.6708 (15) Å, where *Cg*1 and *Cg*3 are the centroids of N1/N2/C5/C10/C11 and C5–C10 rings, respectively.

### Experimental

A mixture of benzimidazole (2.95 g, 25 mmol) and finely ground potassium hydroxide (2.36 g, 30 mmol) in 30 ml of DMSO was stirred at room temperature (27–28 °C) for 30 min. 1-Bromopropane (2.27 ml, 25 mmol) was added drop-wise into this consistently stirred mixture with further stirring for 2 h at the same temperature. The mixture was then poured into water (400 ml) and was extracted by chloroform (5 *x* 20 ml). The extract was dried by magnesium sulfate and evaporated under reduced pressure to get *N*-ethylbenzimidazole (1) as a thick yellowish fluid. Furthermore, a mixture of 1 (1.60 g, 10 mmol) and 1,4-bis(chloromethyl)benzene (0.88 g, 5 mmol) in dioxane (30 ml) was refluxed at 100 °C for 18 h. This desired compound (2.2Cl) appeared as white precipitates in the light brown solution. The mixture was filtered and the precipitates were washed with fresh dioxane (3 × 5 ml), dried at room temperature for 24 h, and the soft lumps obtained were ground into a fine powder (2.15 g, 87%). Saturated solution of 2.2Cl in methanol (0.5 ml) was exposed to diethyl ether vapours (vapour diffusion) at room temperature overnight to get single crystals suitable for X-ray diffraction study.

### Refinement

Atoms H1W1 and H2W1 were located in a difference Fourier map and refined freely. The remaining H atoms were positioned geometrically [C—H = 0.95, 0.98 or 0.99 Å] and refined using a riding model with *U*_iso_(H) = 1.2 or 1.5*U*_eq_(C). A rotating group model was applied to the methyl group.

### Crystal data

**Table:**

C_28_H_32_N_4_^2+^·2Cl^−^·2H_2_O	*F*(000) = 564
*M**_r_* = 531.51	*D*_x_ = 1.305 Mg m^−^^3^
Monoclinic, *P*2_1_/*c*	Mo *K*α radiation, λ = 0.71073 Å
Hall symbol: -P 2ybc	Cell parameters from 5809 reflections
*a* = 8.1177 (5) Å	θ = 2.5–32.5°
*b* = 9.1042 (5) Å	µ = 0.27 mm^−^^1^
*c* = 18.3548 (11) Å	*T* = 100 K
β = 94.323 (2)°	Block, colourless
*V* = 1352.66 (14) Å^3^	0.47 × 0.23 × 0.14 mm
*Z* = 2	

### Data collection

**Table:**

Bruker SMART APEXII CCD area-detector diffractometer	3085 independent reflections
Radiation source: fine-focus sealed tube	2660 reflections with *I* > 2σ(*I*)
Graphite monochromator	*R*_int_ = 0.053
φ and ω scans	θ_max_ = 27.5°, θ_min_ = 2.2°
Absorption correction: multi-scan (*SADABS*; Bruker, 2009)	*h* = −10→10
*T*_min_ = 0.882, *T*_max_ = 0.962	*k* = −11→11
11942 measured reflections	*l* = −22→23

### Refinement

**Table:**

Refinement on *F*^2^	Primary atom site location: structure-invariant direct methods
Least-squares matrix: full	Secondary atom site location: difference Fourier map
*R*[*F*^2^ > 2σ(*F*^2^)] = 0.061	Hydrogen site location: inferred from neighbouring sites
*wR*(*F*^2^) = 0.196	H atoms treated by a mixture of independent and constrained refinement
*S* = 1.08	*w* = 1/[σ^2^(*F*_o_^2^) + (0.1065*P*)^2^ + 2.1866*P*] where *P* = (*F*_o_^2^ + 2*F*_c_^2^)/3
3085 reflections	(Δ/σ)_max_ < 0.001
172 parameters	Δρ_max_ = 0.86 e Å^−^^3^
0 restraints	Δρ_min_ = −0.48 e Å^−^^3^

### Special details

**Table:**

Experimental. The crystal was placed in the cold stream of an Oxford Cryosystems Cobra open-flow nitrogen cryostat (Cosier & Glazer, 1986) operating at 100.0 (1) K.
Geometry. All e.s.d.'s (except the e.s.d. in the dihedral angle between two l.s. planes) are estimated using the full covariance matrix. The cell e.s.d.'s are taken into account individually in the estimation of e.s.d.'s in distances, angles and torsion angles; correlations between e.s.d.'s in cell parameters are only used when they are defined by crystal symmetry. An approximate (isotropic) treatment of cell e.s.d.'s is used for estimating e.s.d.'s involving l.s. planes.
Refinement. Refinement of *F*^2^ against ALL reflections. The weighted *R*-factor *wR* and goodness of fit *S* are based on *F*^2^, conventional *R*-factors *R* are based on *F*, with *F* set to zero for negative *F*^2^. The threshold expression of *F*^2^ > σ(*F*^2^) is used only for calculating *R*-factors(gt) *etc*. and is not relevant to the choice of reflections for refinement. *R*-factors based on *F*^2^ are statistically about twice as large as those based on *F*, and *R*- factors based on ALL data will be even larger.

### Fractional atomic coordinates and isotropic or equivalent isotropic displacement parameters (Å^2^)

**Table:**

	*x*	*y*	*z*	*U*_iso_*/*U*_eq_	
Cl1	0.45230 (8)	0.59270 (7)	0.33489 (4)	0.0240 (2)	
O1W	0.3206 (3)	0.8654 (3)	0.23712 (12)	0.0269 (5)	
N1	0.2941 (3)	0.6933 (2)	0.04689 (12)	0.0175 (5)	
N2	0.2605 (3)	0.4951 (2)	0.11124 (12)	0.0179 (5)	
C1	0.0921 (3)	0.9600 (3)	−0.05784 (15)	0.0201 (5)	
H1A	0.1549	0.9329	−0.0974	0.024*	
C2	0.1507 (3)	0.9274 (3)	0.01358 (15)	0.0175 (5)	
C3	0.0581 (3)	0.9677 (3)	0.07154 (15)	0.0201 (5)	
H3A	0.0977	0.9458	0.1203	0.024*	
C4	0.3152 (3)	0.8500 (3)	0.02876 (15)	0.0206 (5)	
H4A	0.3796	0.8579	−0.0148	0.025*	
H4B	0.3786	0.8994	0.0699	0.025*	
C5	0.2927 (3)	0.5759 (3)	−0.00194 (15)	0.0170 (5)	
C6	0.3152 (3)	0.5696 (3)	−0.07626 (15)	0.0194 (5)	
H6A	0.3306	0.6557	−0.1042	0.023*	
C7	0.3140 (3)	0.4312 (3)	−0.10732 (15)	0.0199 (5)	
H7A	0.3285	0.4223	−0.1580	0.024*	
C8	0.2918 (3)	0.3032 (3)	−0.06619 (15)	0.0206 (5)	
H8A	0.2917	0.2104	−0.0898	0.025*	
C9	0.2701 (3)	0.3095 (3)	0.00817 (15)	0.0199 (5)	
H9A	0.2553	0.2235	0.0362	0.024*	
C10	0.2715 (3)	0.4492 (3)	0.03924 (14)	0.0172 (5)	
C11	0.2750 (3)	0.6401 (3)	0.11360 (15)	0.0197 (5)	
H11A	0.2722	0.6979	0.1566	0.024*	
C12	0.2412 (3)	0.3977 (3)	0.17406 (14)	0.0197 (5)	
H12A	0.2731	0.4515	0.2198	0.024*	
H12B	0.3164	0.3125	0.1713	0.024*	
C13	0.0641 (3)	0.3427 (3)	0.17592 (14)	0.0208 (5)	
H13A	0.0267	0.3006	0.1278	0.025*	
H13B	−0.0093	0.4262	0.1856	0.025*	
C14	0.0517 (4)	0.2262 (3)	0.23502 (16)	0.0276 (6)	
H14A	−0.0607	0.1865	0.2324	0.041*	
H14B	0.0779	0.2705	0.2832	0.041*	
H14C	0.1301	0.1468	0.2274	0.041*	
H1W1	0.377 (6)	0.792 (5)	0.264 (2)	0.050 (12)*	
H2W1	0.392 (6)	0.920 (5)	0.220 (2)	0.046 (12)*	

### Atomic displacement parameters (Å^2^)

**Table:**

	*U*^11^	*U*^22^	*U*^33^	*U*^12^	*U*^13^	*U*^23^
Cl1	0.0237 (4)	0.0186 (4)	0.0300 (4)	−0.0012 (2)	0.0043 (3)	−0.0029 (2)
O1W	0.0269 (11)	0.0265 (12)	0.0276 (11)	−0.0050 (9)	0.0037 (8)	−0.0007 (9)
N1	0.0159 (10)	0.0109 (10)	0.0254 (11)	0.0014 (8)	0.0009 (8)	−0.0022 (8)
N2	0.0163 (10)	0.0152 (11)	0.0219 (11)	0.0002 (8)	0.0001 (8)	−0.0025 (8)
C1	0.0189 (12)	0.0129 (12)	0.0289 (14)	−0.0010 (9)	0.0052 (10)	−0.0004 (10)
C2	0.0162 (11)	0.0066 (11)	0.0300 (14)	−0.0004 (8)	0.0041 (9)	−0.0003 (9)
C3	0.0210 (12)	0.0111 (12)	0.0281 (13)	−0.0007 (9)	0.0024 (10)	−0.0003 (10)
C4	0.0173 (12)	0.0119 (12)	0.0328 (14)	0.0002 (9)	0.0032 (10)	0.0003 (10)
C5	0.0116 (11)	0.0110 (12)	0.0280 (13)	0.0000 (8)	−0.0012 (9)	−0.0017 (9)
C6	0.0154 (12)	0.0143 (12)	0.0281 (14)	−0.0004 (9)	−0.0010 (9)	0.0032 (10)
C7	0.0184 (12)	0.0169 (13)	0.0242 (13)	−0.0016 (9)	−0.0004 (9)	−0.0020 (10)
C8	0.0204 (12)	0.0159 (13)	0.0251 (13)	−0.0017 (10)	−0.0010 (9)	−0.0038 (10)
C9	0.0171 (11)	0.0124 (12)	0.0301 (14)	−0.0004 (9)	0.0013 (9)	0.0013 (10)
C10	0.0122 (11)	0.0168 (13)	0.0224 (13)	0.0013 (9)	−0.0010 (9)	−0.0009 (10)
C11	0.0142 (11)	0.0191 (13)	0.0257 (13)	0.0028 (9)	0.0001 (9)	−0.0029 (10)
C12	0.0186 (12)	0.0179 (13)	0.0223 (13)	0.0027 (9)	−0.0013 (9)	0.0042 (10)
C13	0.0175 (12)	0.0221 (14)	0.0227 (13)	0.0023 (10)	0.0017 (9)	−0.0001 (10)
C14	0.0257 (14)	0.0284 (16)	0.0291 (15)	0.0031 (11)	0.0054 (11)	0.0054 (12)

### Geometric parameters (Å, º)

**Table:**

O1W—H1W1	0.93 (5)	C6—C7	1.382 (4)
O1W—H2W1	0.84 (5)	C6—H6A	0.9500
N1—C11	1.337 (3)	C7—C8	1.407 (4)
N1—C5	1.395 (3)	C7—H7A	0.9500
N1—C4	1.478 (3)	C8—C9	1.390 (4)
N2—C11	1.326 (4)	C8—H8A	0.9500
N2—C10	1.395 (3)	C9—C10	1.393 (4)
N2—C12	1.472 (3)	C9—H9A	0.9500
C1—C3^i^	1.392 (4)	C11—H11A	0.9500
C1—C2	1.393 (4)	C12—C13	1.526 (4)
C1—H1A	0.9500	C12—H12A	0.9900
C2—C3	1.397 (4)	C12—H12B	0.9900
C2—C4	1.517 (3)	C13—C14	1.526 (4)
C3—C1^i^	1.392 (4)	C13—H13A	0.9900
C3—H3A	0.9500	C13—H13B	0.9900
C4—H4A	0.9900	C14—H14A	0.9800
C4—H4B	0.9900	C14—H14B	0.9800
C5—C6	1.391 (4)	C14—H14C	0.9800
C5—C10	1.397 (4)		
			
H1W1—O1W—H2W1	107 (4)	C9—C8—C7	121.6 (2)
C11—N1—C5	108.4 (2)	C9—C8—H8A	119.2
C11—N1—C4	125.4 (2)	C7—C8—H8A	119.2
C5—N1—C4	126.2 (2)	C8—C9—C10	116.2 (2)
C11—N2—C10	108.5 (2)	C8—C9—H9A	121.9
C11—N2—C12	126.1 (2)	C10—C9—H9A	121.9
C10—N2—C12	125.4 (2)	C9—C10—N2	131.5 (2)
C3^i^—C1—C2	120.2 (2)	C9—C10—C5	122.0 (2)
C3^i^—C1—H1A	119.9	N2—C10—C5	106.5 (2)
C2—C1—H1A	119.9	N2—C11—N1	110.3 (2)
C1—C2—C3	119.7 (2)	N2—C11—H11A	124.8
C1—C2—C4	120.4 (2)	N1—C11—H11A	124.8
C3—C2—C4	119.9 (2)	N2—C12—C13	111.8 (2)
C1^i^—C3—C2	120.1 (3)	N2—C12—H12A	109.3
C1^i^—C3—H3A	120.0	C13—C12—H12A	109.3
C2—C3—H3A	120.0	N2—C12—H12B	109.3
N1—C4—C2	112.0 (2)	C13—C12—H12B	109.3
N1—C4—H4A	109.2	H12A—C12—H12B	107.9
C2—C4—H4A	109.2	C12—C13—C14	110.9 (2)
N1—C4—H4B	109.2	C12—C13—H13A	109.5
C2—C4—H4B	109.2	C14—C13—H13A	109.5
H4A—C4—H4B	107.9	C12—C13—H13B	109.5
C6—C5—N1	131.8 (2)	C14—C13—H13B	109.5
C6—C5—C10	121.8 (2)	H13A—C13—H13B	108.1
N1—C5—C10	106.3 (2)	C13—C14—H14A	109.5
C7—C6—C5	116.4 (2)	C13—C14—H14B	109.5
C7—C6—H6A	121.8	H14A—C14—H14B	109.5
C5—C6—H6A	121.8	C13—C14—H14C	109.5
C6—C7—C8	122.0 (2)	H14A—C14—H14C	109.5
C6—C7—H7A	119.0	H14B—C14—H14C	109.5
C8—C7—H7A	119.0		
			
C3^i^—C1—C2—C3	−0.1 (4)	C8—C9—C10—N2	−177.0 (2)
C3^i^—C1—C2—C4	−179.4 (2)	C8—C9—C10—C5	0.2 (4)
C1—C2—C3—C1^i^	0.1 (4)	C11—N2—C10—C9	177.2 (3)
C4—C2—C3—C1^i^	179.4 (2)	C12—N2—C10—C9	−1.1 (4)
C11—N1—C4—C2	−86.9 (3)	C11—N2—C10—C5	−0.3 (3)
C5—N1—C4—C2	94.0 (3)	C12—N2—C10—C5	−178.6 (2)
C1—C2—C4—N1	−103.9 (3)	C6—C5—C10—C9	−0.6 (4)
C3—C2—C4—N1	76.9 (3)	N1—C5—C10—C9	−177.7 (2)
C11—N1—C5—C6	−176.6 (3)	C6—C5—C10—N2	177.2 (2)
C4—N1—C5—C6	2.6 (4)	N1—C5—C10—N2	0.1 (3)
C11—N1—C5—C10	0.1 (3)	C10—N2—C11—N1	0.3 (3)
C4—N1—C5—C10	179.3 (2)	C12—N2—C11—N1	178.6 (2)
N1—C5—C6—C7	176.8 (2)	C5—N1—C11—N2	−0.3 (3)
C10—C5—C6—C7	0.6 (4)	C4—N1—C11—N2	−179.5 (2)
C5—C6—C7—C8	−0.2 (4)	C11—N2—C12—C13	104.6 (3)
C6—C7—C8—C9	−0.1 (4)	C10—N2—C12—C13	−77.4 (3)
C7—C8—C9—C10	0.1 (4)	N2—C12—C13—C14	172.3 (2)

Symmetry code: (i) −*x*, −*y*+2, −*z*.

### Hydrogen-bond geometry (Å, º)

**Table:**

*D*—H···*A*	*D*—H	H···*A*	*D*···*A*	*D*—H···*A*
O1*W*—H1*W*1···Cl1	0.93 (4)	2.29 (4)	3.200 (3)	166 (4)
O1*W*—H2*W*1···Cl1^ii^	0.84 (5)	2.30 (4)	3.130 (3)	170 (4)
C1—H1*A*···Cl1^iii^	0.95	2.81	3.677 (3)	153
C4—H4*B*···Cl1^ii^	0.99	2.77	3.741 (3)	167
C6—H6*A*···Cl1^iii^	0.95	2.76	3.691 (3)	165
C11—H11*A*···O1*W*	0.95	2.14	3.059 (4)	163
C12—H12*A*···Cl1	0.99	2.79	3.747 (3)	164
C12—H12*B*···Cl1^iv^	0.99	2.75	3.740 (3)	175

Symmetry codes: (ii) −*x*+1, *y*+1/2, −*z*+1/2; (iii) *x*, −*y*+3/2, *z*−1/2; (iv) −*x*+1, *y*−1/2, −*z*+1/2.
